# Impact of Diabetes on Cardiovascular Disease: An Update

**DOI:** 10.1155/2013/653789

**Published:** 2013-03-04

**Authors:** Alessandra Saldanha de Mattos Matheus, Lucianne Righeti Monteiro Tannus, Roberta Arnoldi Cobas, Catia C. Sousa Palma, Carlos Antonio Negrato, Marilia de Brito Gomes

**Affiliations:** ^1^Department of Internal Medicine, Diabetes Unit, State University of Rio de Janeiro, Avenida 28 de Setembro 77, Terceiro Andar, Vila Isabel, 20551-030 Rio de Janeiro, RJ, Brazil; ^2^Department of Internal Medicine, Bauru's Diabetics Association, Rua Saint Martin 27-07, 17.012-433 Bauru, SP, Brazil

## Abstract

Cardiovascular diseases are the most prevalent cause of morbidity and mortality among patients with type 1 or type 2 diabetes. The proposed mechanisms that can link accelerated atherosclerosis and increased cardiovascular risk in this population are poorly understood. It has been suggested that an association between hyperglycemia and intracellular metabolic changes can result in oxidative stress, low-grade inflammation, and endothelial dysfunction. Recently, epigenetic factors by different types of reactions are known to be responsible for the interaction between genes and environment and for this reason can also account for the association between diabetes and cardiovascular disease. The impact of clinical factors that may coexist with diabetes such as obesity, dyslipidemia, and hypertension are also discussed. Furthermore, evidence that justify screening for subclinical atherosclerosis in asymptomatic patients is controversial and is also matter of this review. The purpose of this paper is to describe the association between poor glycemic control, oxidative stress, markers of insulin resistance, and of low-grade inflammation that have been suggested as putative factors linking diabetes and cardiovascular disease.

## 1. Introduction 

Diabetes is an important chronic disease which incidence is globally increasing and though considered as an epidemic [1]. The World Health Organization (WHO) estimated there were 30 million people who had diabetes worldwide in 1985. This number increased to 135 million by 1995 and reached 217 million in 2005. By the year 2030 WHO predicts this number will increase to at least 366 million [1]. This growth in diabetes prevalence, driven principally by an increased prevalence of type 2 diabetes (T2D), is occurring in both developing and developed countries [1]. The incidence of type 1 diabetes (T1D) is also increasing in parallel to that of T2D worldwide [2–4].

Individuals with diabetes and with chronically poor metabolic control can experience microvascular and macrovascular complications leading to a significant burden for the individual and for the society. This burden includes direct costs of medical care and indirect costs, such as loss of productivity, which result from diabetes-related morbidity and premature mortality [5, 6]. Health care expenses for people with diabetes is more than double of that for people without diabetes; the direct and indirect expenditures attributable to diabetes in 2007 in the USA were conservatively estimated at $174 billion, with slightly more spent on chronic complications attributable to diabetes than on diabetes care itself [6]. The International Diabetes Federation (IDF) estimated that diabetes accounts for 5–10% of the total healthcare budget in many countries [3]. The outpatient costs of T2D in Brazil were estimated by the ESCUDI study in 2011 [7]. The total costs were US$ 2,108 per patient/year, which consisted mostly of direct costs (63.3%) [7].

 Cardiovascular diseases (CVD) are the most prevalent cause of mortality and morbidity among people with T2D and T1D [8–10]. In 2004, in the USA the presence of CVD and stroke was found in 68% and 16% of deaths related to diabetes among people older than 65 years, respectively [11]. Adult people with diabetes present rates of mortality due to heart disease and stroke from two to four times higher than those without diabetes [11]. It has been stated that patients with T2D without a previous history of myocardial infarction have the same risk of coronary artery disease (CADs) as nondiabetic subjects with a history of myocardial infarction [12]; this has led the National Cholesterol Education Program to consider diabetes as a coronary heart disease risk equivalent [13]. However, there is still some uncertainty as to whether the cardiovascular risk conferred by diabetes is truly equivalent to that of a previous myocardial infarction [14]. In general, patients with diabetes aggregate other comorbidities such as obesity, hypertension, and dyslipidemia which also contribute to increase the risk for CVD [15]. In the period of 2005 to 2008, the American Diabetes Association (ADA) estimated that 67% of people with diabetes older than 20 years presented blood pressure levels ≥140/90 mmHg or were using antihypertensive drugs [16]. Although there is strong evidence that supports both the efficacy and cost effectiveness of programs directed towards an improvement of glycemic control and other cardiovascular risk factors in patients with T2D [17] and T1D [18], the majority of these patients [19, 20] never achieve the goals established by guidelines issued by diabetes societies [16, 21].

 The underlying mechanisms that cause accelerated atherosclerosis in patients with diabetes and consequently an increased prevalence of CVD are poorly understood. The purpose of this paper is to describe the association between poor glycemic control, oxidative stress, markers of insulin resistance, and of low-grade inflammation that have been suggested as putative factors linking these two conditions.

## 2. The Role of Glycemic Control

In recent decades, several clinical trials have investigated the effect of intensive treatment of hyperglycemia on cardiovascular risk reduction, in both T2D [22–25] and T1D [26] presenting conflicting results. The clinical characteristics of the studied populations regarding the presence of CVD and the duration of diabetes as well as the type of intensive intervention performed and the goals to be achieved partly explain the differences in the results. 

 In the *United Kingdom Prospective Diabetes Study* (UKPDS) [22], in newly diagnosed T2D patients, the early intensive treatment of hyperglycemia within the first five years of disease resulted in a long-term cardiovascular benefit, compared with patients in the conventional treatment group. This benefit was observed even after the loss of difference in glycemic control between the groups that occurred during the further five years of observational followup. However, the same was not observed in the three other large clinical trials conducted in patients with T2D. In the *Veterans Affairs Diabetes Trial* (VADT) [23], older patients with a 10 years mean duration of diabetes had no cardiovascular benefit when submitted to an intensive glycemic control regimen. This population comprised 40% of patients with a previous history of cardiovascular disease. Similar results were obtained from the *Action in Diabetes and Vascular Disease: Preterax and Diamicron Modified Release Controlled Evaluation* (ADVANCE) trial [24] which aimed to achieve an A1c of 6.5% through intensive treatment with gliclazide plus other drugs. This strategy did not reduce the rate of major macrovascular events or death, despite a reduction in the incidence of diabetic nephropathy. As in VADT, patients were older (mean age of 66 years) and had a longer duration of diabetes (8 years) than UKPDS patients when the intensive treatment was started. In contrast, more strict intensive treatment aiming to reduce HbA1c below 6% in T2D patients, as occurred in the *Action to Control Cardiovascular Risk in Diabetes *(ACCORD) trial [25], in addition to showing no benefit in reducing macrovascular events resulted in increased mortality, weight gain, and risk of hypoglycemia. Patients in this study presented a high cardiovascular risk profile when first initiated intensive glycemic treatment.  Table 1 presents the main differences between these trials.

 In T1D patients, *the Diabetes Control and Complications Trial/Epidemiology of Diabetes Interventions and Complications* (DCCT/EDIC) study [26] showed the cardiovascular benefits of an intensive glycemic control after a followup of 17 years. The patients in this study were treated intensively for about 6.5 years and followed for 10 years observationally. Even after losing the strict glycemic control, represented by a glycated hemoglobin level below 7%, during the observational period, the group previously intensively treated presented a reduction of any cardiovascular event by 42%.

The main lesson learned from these results is that intensive treatment of hyperglycemia, targeting glycated hemoglobin levels below 7%, when initiated early in patients with short duration of diabetes and low cardiovascular risk, results in cardiovascular benefits. The same is not true when looking up tighter glycemic targets in older patients exposed to hyperglycemia for years before and with a higher cardiovascular risk profile. 

**Figure 1:**
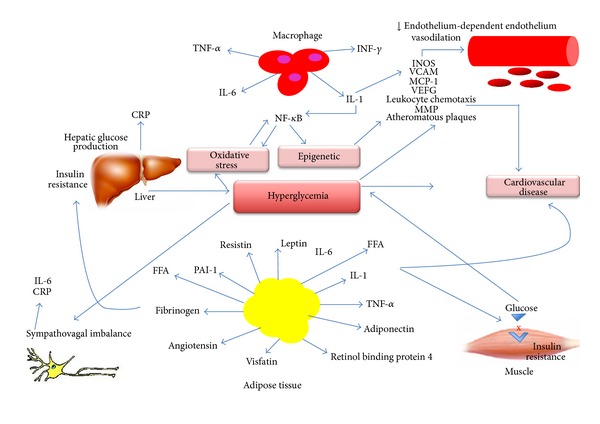
Pathogenesis of cardiovascular disease in diabetes. The mechanisms involved in the pathogenesis of cardiovascular disease in diabetes comprehend epigenetic changes and intracellular metabolic changes that result in oxidative stress, low-grade inflammation, and endothelial dysfunction. CRP: C-reactive protein; FFA: free fatty acids; INOS: inducible nitric oxide synthase; IL-1: interleukin 1; IL-6: interleukin 6; MCP-1: monocyte chemoattractant molecule 1; MMP: matrix metalloproitenase; NF-*κ*B: nuclear factor kappa-*β*; PAI-1: plasminogen activator inhibitor-1; VCAM-1; vascular cell adhesion molecule-1; VEFG: vascular endothelial growth factor; TNF-*α*: Tumor necrosis factor-*α*; INF-*γ*: Interferon-*γ*.

This early protection is postulated to result from a mechanism known as “metabolic memory,” which means that the effect of the early glycemic exposure environment is remembered later in target organs [27] resulting in long-term deleterious or protective effects. The mechanisms involved in this process appear to comprehend epigenetic changes and intracellular metabolic changes that result in oxidative stress, low-grade inflammation, and endothelial dysfunction (Figure 1). These topics will be discussed below.

## 3. Obesity

 Obesity, which prevalence is also increasing worldwide [28] is becoming a major public health issue due to its association with chronic diseases such as diabetes mellitus, hypertension, dyslipidemia, sleep apnea, osteoarticular disease, and cardio and cerebrovascular diseases [28]. According to data from the WHO in 2008, the global prevalence of obesity (body mass index (BMI) ≥ 30 kg/m^2^) was 10% in men and 14% in women. Data from the National Health and Nutrition Examination Survey (NHANES) showed that the prevalence of overweight and obesity in adults increased from 55.9% to 64.5% and from 22.9% to 30.5%, from 1988–1994 to 1999-2000, respectively [28].

 Obesity, especially with visceral fat deposition, is associated with low-grade inflammation, which plays a role in the pathogenesis of diabetes, and both diseases are associated with significant increase in morbidity and mortality due to CVD [28–30]. 

The main determinants for the onset of diabetes are beyond genetic factors, obesity and sedentary lifestyle [31]. Several studies have shown decreased incidence of diabetes by nonpharmacologic treatments, lifestyle changes, and body weight reduction. The Finish Diabetes Prevention Study Group showed that the incidence of diabetes was reduced in 58% in the group with only intensive lifestyle changes [31]. The Diabetes Prevention Program (DPP), diabetes incidence was reduced by 58% with intensive lifestyle intervention when compared to placebo and remained reduced by 34% after 10 years of followup [32]. Therefore, efforts should be made to encourage the adoption of healthy lifestyle and thus to combat the obesity epidemic.

## 4. Dyslipidemia

 Dyslipidemia in T2D worsens cardiovascular risk due to the peculiar atherogenic profile composed by increased very low-density lipoprotein (VLDL) cholesterol, triglycerides and small and dense LDL cholesterol levels and decreased high-density lipoprotein (HDL) cholesterol levels. With such lipoproteins modified by oxidation and glycosylation there is a reduction on vascular compliance predisposing to early and aggressive atherosclerosis [33]. This may also occur in T1D, even though they are young patients and seldom present lipid abnormalities, but in this case, the atherogenic profile is not caused exclusively by increased lipid levels, and hyperglycemia *per se* is also pivotal in this process [34]. This was evidenced in an experimental study which concluded that either diabetic hyperlipidemia or hyperglycemia accelerates distinct phases of atherogenesis in diabetes [35]. In this study, it was shown that the dyslipidemia associated with diabetes is not sufficient to initiate the atherosclerotic lesion, because the progression of atherosclerosis process could be normalized after intensive glycemic control with insulin in mice [35].

 In many interventional studies, the reduction of LDL cholesterol and triglycerides and increase of HDL cholesterol have been proved to be effective in reducing macrovascular disease and mortality in patients with T2D, especially in those with previous CAD.

 The *Collaborative Atorvastatin Diabetes Study *(CARDS) was the first trial that studied T2D patients without previous CVD. Intervention with atorvastatin 10 mg showed 37% reduction in Cardiovascular events and 48% reduction in stroke when compared to placebo [36]. In the *HDL Atherosclerosis Treatment Study* (HATS), the combined use of low doses of simvastatin (10 to 20 mg/day) with high doses of niacin (2 to 4 g/day) showed a reduction in absolute risk of 13% for cardiovascular outcomes when HDL reached the target [37]. The study TNT (treatment to new targets) studied patients with T2D with previous CVD and compared the use of Atorvastatin 10 mg (conventional group) with Atorvastatin 80 mg (intensive group), and the goals were 100 mg and 80 mg for LDL cholesterol, respectively. The aggressive target achieved in this study (1.9 mmol/L) showed the most reduced rates of mortality due to cardiovascular events among all studies with statins [38].

 Although decreasing LDL cholesterol has brought enough and established evidence on reducing cardiovascular mortality in T2D, if the treatment of dyslipidemia starts too late it may not be effective in avoiding atherosclerosis progression. According to the Deutsche Diabetes Dialyze Study (4D) that studied 1,255 T2D patients with end-stage renal disease which were randomized to Atorvastatin 20 mg/day or matching placebo during four years, there was no significant reduction in cardiovascular events with the intervention when compared to placebo [39]. Concerning cholesterol goals for diabetics, as far as we know, we should get as lower cholesterol levels as possible as stated by National Cholesterol Education Program Adult Treatment Panel III Guidelines (NCEP ATP III) [40]. It is well established that diabetic subjects are considered to belong to a high-risk category, thus their benefit from LDL-lowering therapy appears when LDL-C goal of 1.8 mmol/L is achieved [40].

 In a recent meta-analysis which reviewed 22 trials with statins versus control, it was showed that statin use could be associated with an increased incidence of diabetes [40]. Despite the fact that an immediate doubling in cardiovascular risk in individuals with 5-year risk of major vascular events lower than 10%, such an effect is more than 50-times smaller than the absolute benefit observed with statin therapy in such individuals (about 11 fewer major vascular events per 1,000 treated over 5 years per 1.0 mmol/L reduction in LDL cholesterol) [41].

 Considering hypertriglyceridemia, there is little evidence to support the benefits the goals to be achieved can bring. Fibrates are recommended to reduce pancreatitis risk in patients with triglycerides levels above 4.5 mmol/L when lifestyle modification does not succeed [42]. Until recently, there were no data that support that the combined use of statins and fibrates could reduce cardiovascular mortality. The Fenofibrate Intervention and Event Lowering in Diabetes (FIELD) study was a multinational randomized trial conducted with 9,795 patients with T2D which showed that fenofibrate did not significantly reduce the risk of the primary outcomes of coronary events. Instead, it reduced the number of total cardiovascular events (fewer nonfatal myocardial infarctions and revascularizations) [43]. Another message from this study was that fibrate confers microvascular protection, because it reduced the need for laser treatment for diabetic retinopathy [44].

## 5. Hypertension

Hypertension is a highly prevalent disease worldwide and very common among patients with diabetes. Approximately from 10 to 30% of T1D and 60% of T2D patients have hypertension [45, 46].

The coexistence of these two conditions increase the risk of developing macrovascular complications (myocardial infarction, stroke) and also microvascular complications (nephropathy and retinopathy) [47]. The vigorous treatment of hypertension may reduce the progression of these complications.

The time hypertension starts differs in different types of diabetes. In patients with T1D, hypertension develops years after diagnosis usually already reflecting the development of diabetic nephropathy [46]. Blood pressure (BP) tends to increase three years after the onset of microalbuminuria [48]. In patients with T2D, hypertension may be present at diagnosis or even before the elevation of blood glucose levels [49]. The association between hypertension and obesity is well established leading to a higher rate of cardiovascular morbidity and mortality in patients with these two conditions [49].

The recommended target blood pressure for patients with diabetes, according to the ADA [50] is characterized by BP < 130/80 mmHg [51]. Although, the European Society of Hypertension Task Force [52] states that BP goals traditionally recommended in diabetes are not supported by outcomes evidence from trials. They also reinforce only to pursue a reasonable BP reduction without indicating a goal which is unproven, since it has also been very difficult to achieve blood pressure goals in the majority of the patients [53].

According to the ADVANCE study in diabetic patients at high cardiovascular risk lower BP levels should be reached [53]. One should always take into consideration the individualization of treatment and its correlation with response to therapy, drug tolerance, and individual characteristics. However, randomized clinical trials have demonstrated that the established therapeutic target (BP < 130/80 mmHg) own benefits in reducing CHD, stroke, and kidney disease [52, 54]. In patients with renal insufficiency and proteinuria above 1 to 2 g per day, the target BP should approach 120/75 mmHg [53].

The treatment of hypertension in diabetic patients aims at the prevention of CVD, minimizing the progression of renal disease and diabetic retinopathy. According to the UKPDS, patients with T2D may benefit more from tight control of BP than with strict control of blood glucose levels [55]. Initial treatment should include nonpharmacological measures such as weight reduction (in overweight and obesity), regular exercising, reducing salt intake (<1500 mg per day), avoiding excessive alcohol consumption (no more than two servings per day in men and no more than one serving per day in women), and smoking cessation. Pharmacological therapy should be initiated in all diabetics who persist with BP > 130/80 mmHg, when a change in lifestyle has already been implemented for 3 months or when the maximum BP levels are already higher than 140/90 mmHg at diagnosis [50, 56].

Pharmacological therapy can be accomplished with various classes of antihypertensive agents. Diuretics, angiotensin converting enzyme inhibitors, angiotensin II antagonists, beta blockers, calcium channel blockers, alpha blockers, and combination of blockers of the renin-angiotensin have shown to be effective in reducing cardiovascular events. In most cases, the association of two or three drugs may be necessary in order to achieve the goals of the treatment.

The ACCORD-BP study, evaluating more intensive treatment of blood pressure (systolic blood pressure reduction aiming at levels lower than 120 mmHg) in patients with T2D and CVD or at least two cardiovascular risk factors, showed no reduction in cardiovascular events rates (myocardial infarction, CHF, and cardiovascular death), although it was observed a reduction in the number of strokes [57].

## 6. Oxidative Stress

Increased intracellular glucose concentrations result in the activation of alternative pathways of metabolism such as the hexosamine and the aldose reductase pathways, both involved in the pathophysiology of chronic complications of diabetes. These pathways trigger an increased production of reactive oxygen species (ROS) and depletes substrates for important antioxidant enzymes. Additionally, increased intracellular glucose leads to the formation of advanced glycation end products (AGES) and the activation of protein kinase C (PKC). All these mechanisms lead to a common effect, an increased oxidative stress state.

 Oxidative stress results from an imbalance between the production of ROS and the antioxidant defense. The ROSs are chemically instable and highly reactive molecules [58] continuously produced by aerobic organisms that function as second messengers regulating the expression of redox signal sensitive genes (e.g., nuclear factor kappa-*β* (NF*κ*-B) gene) and in the production of inflammatory mediators. They are generated from enzymes that use oxygen as electron acceptor including the nicotinamide adenine dinucleotide phosphate (NADPH) oxidase, nitric oxide synthase (NOS), xanthine oxidase, the mitochondrial chain electron transport, lipoxygenase, cyclooxygenase, and cytochrome P450. The first three are the main sources of ROS in the vascular wall [59].

 The active form of NADPH oxidase is responsible for the reduction of the molecular oxygen resulting in the formation of superoxide anion [NAD(P)H + 2O_2_ → NAD(P)^+^ + H^+^ + 2O_2_^−^] [58]. This enzyme could act as a sensor of the concentration of oxygen in the vasculature modulating the vascular tone [60]. Components of the NADPH oxidase were demonstrated in vascular and renal cells in animals and humans [61–67].

The ROSs produced in the vascular wall are involved in various cellular events such as mitosis, apoptosis, migration, hypertrophy and extracellular matrix modification, and changes in gene transcription and protein synthesis [68]. They may also function as mediators of the metabolic memory to hyperglycemia. Human retinal endothelial cells exposed to hyperglycemia in vitro, maintained high levels of oxidative stress markers such as PKC and *β* subunits of NADPH oxidase p47phox, even after normalization of blood glucose levels [69].

 Another important source of ROS in diabetes is the mitochondria. It is postulated that the mitochondrial O_2_^−^ anion acts as a factor initiating a cascade of events that result in increased production of ROS and reactive nitrogen species (RNS) through activation of NF*κ*-B. This results in the production of inflammatory cytokines, activation of PKC and NADPH oxidase. In addition, NOS can divert the production of nitric oxide (NO) to generate O_2_^−^ in conditions of deficiency of L-arginine or tetrahydropterin in the endothelium of diabetic patients [70]. When both are produced the formation of peroxynitrite (NOO^−^) occurs, causing damage to cellular structures such as DNA, lipids, and proteins [71].

Under normal conditions, the presence of ROS induces the expression of antioxidant enzymes as a defense mechanism. This is not a rule under diabetes condition. For instance, in fibroblasts from T1D patients with overt nephropathy, the exposure to hyperglycemia led to an increase in lipid peroxidation without a compensatory increase in the level of the antioxidant enzyme Cu-Zn superoxide dismutase, catalase, and glutathione peroxidase [72]. Even patients with a short diabetes duration and without chronic complications present less antioxidant plasma capacity and uric acid levels suggesting that the oxidative stress occurs early in the disease [73]. 

 Nonenzymatic extracellular antioxidants include *α*-tocopherol, vitamin A, *β*-carotene, ascorbic acid, albumin, and uric acid. The lipid solubility properties of *α*-tocopherol, vitamin A, and *β*-carotene are particularly important to protect against lipid peroxidation. The role of uric acid in the pathogenesis of CVD and endothelial dysfunction is still conflicting [74–76]. Another important component of the antioxidant defense in diabetes is haptoglobin [77–79]. This plasma protein binds free hemoglobin resulting in the inhibition of iron-induced oxidative damage, since hemoglobin released in the blood after hemolysis of senescent erythrocytes is a potent oxidant.

## 7. Epigenetics

 Nowadays, there is compelling evidence linkingepigenetic factors to many human diseases including diabetes and CVD [80]. Epigenetic factors, by different types of reactions, could mediate the interplay between genes and environment resulting in activation or repression of genetic transcription, or even silencing the genetic transcription. The most important epigenetic reactions affecting genetic transcription are acetylation and methylation. These reactions occur mainly in the tail of histones that are proteins where DNA is wrapped. Brownlee et al. [81] have demonstrated in human aortic endothelial cells that excess ROS resulting from hyperglycemia can induce monomethylation of lysine from histone 3 increasing the expression of the subunit p65 of NF*κ*-B. This reaction is responsible for the increased transcription of vascular cell adhesion molecule 1 (VCAM-1), monocyte chemoattractant molecule 1 (MCP-1), and some inflammatory proteins like interleukin 6 (IL-6), intercellular adhesion molecule 1 (ICAM-1), and NOS that are related tohyperglycemia-induced arterial pathology. Moreover, this reaction persisted after asix-day period of subsequent normoglycemia, supporting the concept of metabolic memory. Epigenetic reactions could be an important mediator between diabetes, CVD, and chronic inflammatory response. Besides, some comorbidities associated with diabetes have also been associated with epigenetics like hypertension [82] and obesity [83]. The epigenetic modifications associated with hypertension are related to intrauterine environmental factors which can limit the development of the nephrons and to other factors that are related to autonomic responsiveness, vessel remodeling, salt sensitivity, and to the renin-angiotensin system. The mechanisms involved in these associations are mainly methylation of histones and of DNA. The relationship between epigenetics and obesity is more complex and is related to genomic imprinting, epigenetic mosaicism, and nonimprinted gene which through different pathways can influence energy balance, body weight, and fat mass.

## 8. Inflammatory Cascade, Diabetes, and Atherosclerosis

 Diabetes, obesity, and insulin resistance are associated with subclinical inflammation characterized by overexpression of cytokines produced by adipose tissue, activated macrophages, and other cells [84, 85]. Inflammatory mediators, such as TNF-*α*, interleukin-1 (IL-1), IL-6, leptin, resistin, MCP-1, plasminogen activator inhibitor-1 (PAI-1), C-reactive protein (CRP), fibrinogen, angiotensin, visfatin, retinol binding protein-4, and adiponectin are involved in signaling pathways, in insulin action, and perpetuation of inflammatory response [85]. These cytokines are involved in the chronic inflammatory process of the vessels wall, promoting lipid accumulation with consequent development of atherosclerosis and CVD [86].

 Atherosclerosis is a complex multifactorial disease, and the acceleration of atherosclerosis in diabetes may be explained by several conditions including hyperglycemia, increased oxidative stress, advanced glycation end products (AGE), dyslipidemia, autonomic imbalance, hyperinsulinemia, inflammatory markers excess, and genetic variables [35, 87, 88].

It is assumed that the adipose tissue initiates obesity-induced inflammation and leads to the recruitment of immune cells which contributes to the maintenance of inflammatory response [84], besides leading to endothelial dysfunction with increased expression of adhesion molecules (ICAM-1, VCAM-1, P-selectin, and E-selectin), migration of monocytes, neutrophils, and T lymphocytes [89].

Insulin resistance induces chronic elevation in FFA plasma concentrations leading to increased storage of triglycerides in muscle, promoting reduction of muscle glucose uptake and liver, and increased hepatic glucose production, that have been shown to impair insulin action and promote hyperinsulinemia [90]. Hyperinsulinemia can, per se, induce cardiomyocyte hypertrophy through myocyte growth induced by an activation of PI3 K/Akt-1 pathway and also by enhancing FFA levels. FFA are also implicated in the development of myocardial contractile dysfunction [91]. 

Several cytokines described to be related with insulin resistance are also involved with the development of atherosclerosis and CVD. TNF-*α* and other cytokines, FFA and ROS, activate inflammatory pathways and promote the expression of numerous genes involved in insulin resistance [84, 85, 89]. 

IL-1 is another cytokine produced as a consequence of stress or cell injury mainly by macrophages that modulate key events in the process of atherosclerosis such as vessels wall inflammation, leukocyte chemotaxis and adhesion by increasing expression of VCAM-1 and MCP-1, angiogenesis (through vascular endothelial growth factor—(VEFG) induction), upregulation of matrix metalloproteinases (MMP), and destabilization of atheromatous plaques, that can lead to plaque rupture and thrombosis [86].

CRP is an acute phase protein and is primarily derived from IL-6 hepatic biosynthesis [92]. Atherogenic mechanisms of CRP include impaired production of endothelial NO and prostacyclin; increased production of endothelin-1 and other cell adhesion molecules, monocyte chemoattractant protein-1, IL-8, and PAI-1; ROS and proinflammatory macrophage production; monocyte adhesion and chemotaxis; uptake of oxidized low-density lipoprotein (LDL); CRP also stimulates the expression of metalloproteinases, activates NF-*κ*B, and promotes cell proliferation in vascular smooth muscle cells due to upregulation of the angiotensin type 1 receptor [93].

 Adiponectin has many protective actions in the atherosclerosis process due to its inhibition of LDL oxidation, activation of macrophages (via TNF-*α*), reduction of adhesion molecule (VCAM and ICAM), inhibition of proliferation and migration, of smooth cells, and an increased production of NO in endothelial cells [87]. Adiponectin is markedly reduced with increased obesity, and in diabetes [85] and hypoadiponectinemia is associated with an increase in CVD rates [94].

 Leptin is a hormone secreted by adipose tissue and primarily involved in the regulation of energy expenditure and food intake. Plasma leptin concentrations are increased in obese and diabetic patients [95]. Leptin has been shown to participate in the development of atherosclerosis in several ways: inducing oxidative stress; increasing the production of MCP-1, endotelin-1 (ET-1) which leads to cardiomyocyte hypertrophy; promoting migration, proliferation, hypertrophy of vascular smooth muscle cells (VSMC), and vascular cell wall calcification; stimulating platelet aggregation; attenuating cardiomyocyte contractility through increased nitric oxide production, reduction of intracellular calcium, and decreased *β*-adrenergic response [95].

Therefore, evidences suggest that the hypothesis that is low-grade inflammation would be the causal common factor between diabetes, insulin resistance, obesity, and CVD [32].

## 9. Endothelial Dysfunction 

Endothelial vasodilation and vascular reactivity in diabetes are known to be impaired since its early phases [96, 97]. This is explained by the hypothesis that there are changes in endothelial cells function present in the early atherosclerosis lesion [98]. Thus, oxidative stress, inflammation, and endothelial dysfunction are closely correlated in diabetes, because the formers increase vascular endothelial permeability, generating leukocyte adhesion, which is coupled with impairment in endothelial signal transduction and redox-regulated transcription factors [99, 100]. Another possible mechanism to link these conditions is that the impaired endothelium-dependent vasodilation in diabetes is associated with reduced action of NO secondary to its inactivation, and this is a consequence of oxidative stress, rather than decreased NO production from endothelial cells. Moreover, the abnormal metabolism of NO is related to advanced diabetes microvascular complications [99]. Many factors can explain endothelial dysfunction in diabetes such as hyperlipidemia [96, 98], insulin resistance [86, 98, 101], hyperglycemia [98], hyperamylinemia [101], hypertension [101], and hyperhomocysteinemia [101].

## 10. Endothelial Dysfunction in T1D

 Endothelial function of the macro- and microcirculation, which is usually evaluated through the vasodilator response to endothelium-dependent vasodilators or physiological stimuli, is characteristically impaired in patients with T1D [76, 102–104]. The endothelial response to acetylcholine is correlated with diabetes duration, glycemic control, triglycerides, and age [76, 105, 106]. 

Endothelial dysfunction in T1D is an important determinant of inflammatory activity regardless of the presence or absence of complications showing that it can be considered an early marker for CVD [107]. The disturbances in vascular responses can be seen even in children with T1D, as evidenced in studies that showed impaired flow-mediated dilation (FMD) responses and also association with increased carotid artery intima-media thickness in this group [97, 108]. And, as evidenced by Davi et al. [97], this alteration represents an early and, in some cases, a reversible event in the natural history of T1D in children and adolescents because it was noted that in approximately 45% of this population the tissue plasminogen activator (tPA) levels were reversed after 1 year.

 Several markers of endothelial function in T1D have been described such as Von Willebrand factor, thrombomodulin, selectin, PAI-1, Type IV collagen, and tPA, that are so forth indicators of endothelial cell dysfunction when increased. VCAM-1 levels are more markedly increased in patients with T1D with retinopathy when compared with those with micro- or macroalbuminuria only [98]. It has been shown that the cellular adhesion molecule E-selectin may enhance CAD prediction beyond traditional risk factors in T1D [98, 107]. Other markers of low-grade inflammation levels are described to be elevated in this group such as of oxidized LDL [109], monocyte IL-6, superoxide anion, plasma CRP, sCD40L, and nitrotyrosine levels [110].

So, endothelial dysfunction in T1D represents a high risk for micro- and macroangiopathy and hyperglycemia, appears to be one of the main causes, that alone seems not to be sufficient to cause it, because other agents such as genes and environmental factors are likely to play a role [76, 98].

## 11. Endothelial Dysfunction in T2D

 T2D is independently associated with impaired FMD, and endothelial dysfunction is considered to be the determinant factor for the vascular complications that is aggravated, rather than caused by hyperglycemia, because of the presence of many other risk factors such as obesity, hypertension, dislypidemia, and ageing as well [96, 101, 111]. One possible explanation for this is the increased calpain (calcium-dependent protease) activity in response to hyperglycemia. Hyperglycemic states can induce loss of NO via a calpain-dependent decrease in the association with endothelial NOS. Moreover, inhibition of calpain activity decreases endothelial cell surface expression of the proinflammatory adhesion molecules ICAM-1 and VCAM-1 during hyperglycemia [112]. Markers of endothelial dysfunction are early signs for the development of microangiopathy. 

 The hallmark of T2D is insulin resistance, therefore there is sufficient evidence pointing to the coexistence of endothelial dysfunction with this condition [101]. Elevated circulating levels of PAI-1 and ET-1 can be seen in obesity as well as the correlation between endothelial activation and acute-phase reaction with insulin resistance and obesity in T2D. Abnormalities in vascular reactivity and insulin resistance can also be seen in young first-degree relatives of T2D patients independent of the presence of classic cardiovascular risk factors [113].

## 12. Cardiovascular Autonomic Neuropathy (CAN)

CAN is one of the most common chronic complications of diabetes mellitus and has shown negative impact on survival and quality of life in patients with diabetes [114]. The prevalence of CAN ranges from 2.6% to 90% among subjects with diabetes, and the incidence of CAN increases with age, diabetes duration, and inadequate glycemic control [115]. 

Recent studies [116, 117] have shown that dysregulation of the Autonomic Nervous System (ANS) with increased sympathetic activity is associated with elevated inflammatory markers such as IL-6 and CRP, demonstrating a link between autonomic imbalance, inflammation, and CVD. The ANS is responsible for modulating the activity of the sinus node (heart rate), ventricular (end systolic and diastolic volume) and blood vessels (systemic vascular resistance), and the dysfunction of the ANS may contribute to the development of arterial stiffness, left ventricular hypertrophy, and ventricular diastolic dysfunction [118].

The clinical manifestations of CAN are described as resting tachycardia, postural hypotension, exercise intolerance, abnormal coronary vasomotor regulation (risk of silent myocardial ischemia and infarction), increased QT interval, perioperative instability, increased risk of renal disease, stroke, and sudden death [114].

CAN represents a strong indicator of cardiovascular risk in both T1D and T2D [119]. In the Detection of Silent Myocardial Ischemia in Asymptomatic Diabetic Subjects (DIAD) study, the strongest predictors for abnormal perfusion tests were abnormal Valsalva maneuver, male sex, and diabetes duration, demonstrating that CAN may have an important role in the screening of CVD [120]. Patients with diabetes and CAN have 5-year mortality rates ranging from 16 to 53%, depending on its severity [119]. The mortality rates from CVD in T1D and T2D are 4.2 and 10 times higher, respectively, than in healthy individuals without diabetes [120, 121].

## 13. Screening for Subclinical Atherosclerosis

The screening for the detection of subclinical atherosclerosis in asymptomatic diabetic patients is the subject of considerable controversy. There are no prospective studies that support its usefulness and that can modify the natural history of those patients [122, 123]. Even today, there is no consensus on which tests should be performed. Intensive medical therapy seems to provide equal outcomes to invasive revascularization [124]. Which raises questions on how screening results would change management? The clinical risk factors that indicate increased risk of CVD in diabetic patients are CAD, cerebrovascular or peripheral vascular disease, female sex, age greater than 40 years in men and greater than 50 years in women, long duration of diabetes (for every 10 years the risk increases 86% according to the Framingham study), presence of renal disease, autonomic neuropathy and classic risk factors such as hypertension, dyslipidemia, smoking, sedentary lifestyle, family early atherosclerotic disease, metabolic syndrome, and presence of atrial fibrillation [124, 125].

One of the major limitations of the routine screening for subclinical atherosclerosis is the different rates of coronary events in previous studies. The prevalence of silent myocardial ischemia (SMI) in diabetic population varies in different studies, ranging from 12% to almost 57% [126, 127]. This variability underlines the difficulty to have a cost-effective screening and the necessity to define the cardiovascular risk in the asymptomatic diabetic population who could benefit from this screening. The ADA does not recommend the detection of CVD in asymptomatic diabetic patients as a routine. Their recommendations for investigating SMI are very conservative, being the exercise testing in diabetic patients with typical (chest pain, dyspnea) or atypical cardiac symptoms and changes in baseline electrocardiogram. Asymptomatic patients with carotid or peripheral vascular disease or sedentary patients who want to start high-intensity exercise can also be investigated [50]. The DIAD study [120] accessed 1,123 asymptomatic diabetic patients in a randomized controlled trial. The patients were randomly assigned to be screened with adenosine-stress radionuclide myocardial perfusion imaging (MPI) or not to be screened. The cumulative cardiac event rate was 2.9% over a mean (SD) followup of 4.8 years for an average rate of 0.6% per year. A comparison of the cardiac event rates (0.6% per year) with those reported in ACCORD trial for the subgroup of patients with T2D without previous cardiac events (1.4% per year) which included a selection of older patients with specific additional risk factors for CVD would appear favorable and compatible in these two studies [25]. The data from these two studies show that there is no evidence that the complete survey of subclinical arterial disease may modify the natural history of CAD in asymptomatic diabetic patients with risk factors controlled by recommended goals.

Despite the controversy regarding the screening, several studies using various invasive and noninvasive cardiovascular examinations are being conducted. The presence of calcium in coronary arteries is a specific marker of atherosclerosis, independent of its etiology [128]. The presence of calcified plaques correlates with increasing age, especially after age 50 [129, 130]. Though the calcium score represents an estimate of the total amount of plaque present in an individual, it does not correspond directly to the degree of luminal narrowing of a given vessel [128]. The calcium score was higher than the scores of Framingham and UKPDS for the prediction of events [129]. According to the Patients with Renal Impairment and Diabetes undergoing Computed Tomography (PREDICT) study, the coronary artery calcium (CAC) score was taken as independent risk marker for incremental coronary events and stroke [130].

The US National Cholesterol Education Programme Adult Treatment Panel III (NCEP ATP III) recommends the use of calcium score in a selection of patients with intermediate risk by traditional methods (between 10 and 20% risk in 10 years), and when added to conventional methods, these patients may become high risk, and benefit of a therapy aimed to more restrictive treatment targets [13]. A recently meta-analysis showed that diabetic patients without a history of myocardial infarction had 43% less risk of developing coronary events when compared with patients without diabetes but with prior infarction [14]. Both coronary calcifications as average intimal thickness are increased in this population; however, the classification of these individuals to a higher category of risk is still controversial when using these methods [131]. Although being very promising the use of the calcium score for CVD in asymptomatic diabetic patients still needs further prospective studies and cost effectiveness to demonstrate its benefits.

Revascularization of asymptomatic T2D subjects is still polemic. The Bypass Angioplasty Revascularization Investigation 2 Diabetes Trial (BARI 2D) was a randomized study with 2,368 patients with T2D and SMI comparing revascularization versus intensive medical therapy, showed no differences on reducing rates of death and cardiovascular events among patients undergoing prompt revascularization and those undergoing medical therapy or between strategies insulin sensitizers or insulin provision [123].

According to the 2010 American College of Cardiology Foundation/American Heart Association (ACCF/AHA) Guideline for Assessment of Cardiovascular Risk in Asymptomatic Adults [132], in asymptomatic adults with diabetes, 40 years and older, measurement of CAC score is reasonable for cardiovascular risk assessment (Class IIa, evidence B). Stress MPI may be considered for advanced cardiovascular risk assessment in asymptomatic adults with diabetes or when previous risk assessment testing suggests a high risk of CHD, such as CAC score of 400 or greater (Class IIb, evidence C).

Carotid intima-media thickness (C-IMT) is considered one of the independent predictors of coronary artery disease, a marker of early atherosclerosis and vascular remodeling [133]. According to Irie et al. in an evaluation of 251 asymptomatic T2D patients, the addition of max-IMT (the greatest IMT in the observation-possible areas) to conventional risk factors improves the risk stratification for CAD [133]. In T1D patients, the DCCT/EDIC research group demonstrated that in 12 years after the DCCT intervention the C-IMT progression in the group that received intensive diabetes therapy was slower than the group that received conventional therapy from years 1 to 6. It could be assigned to a durable “metabolic memory” that exists for atherosclerosis. But, the similar C-IMT progression in the original treatment groups over EDIC from years 6 to 12 indicates a “metabolic memory amnesia” over time [134].

The ankle brachial pressure index (ABPI) is a simple method to evaluate the presence of peripheral vascular diseases [135]. A low ABPI (<0.9) was considered to be a marker of cardiovascular diseases risk. The AHA recommends the evaluation of ABPI as a diagnostic criterion for the prevalence of peripheral arterial diseases [136]. In the study of Doza et al. with 1,121 T2D patients in north India, the prevalence of low ABPI was 4.5% in men and 4.7% in woman. The results were similar to those found in studies with Chinese Korean and Brazilian populations [137].

Changing the natural history of silent coronary artery disease, without considering the control of classical risk factors, represents a major challenge facing the global epidemic of diabetes mellitus. More research is needed to identify appropriate screening strategies for diabetic asymptomatic patients with CHD. 

## 14. Perspectives and Conclusions

The incidence of diabetes is sharply increasing worldwide which represents an important burden for patients and for the society as well due to micro- and macrovascular complications that people with this condition may experience and consequently cardiovascular diseases that are the most prevalent causes of morbidity and mortality among patients with diabetes.

The classical risk factors for the development of CVD in subjects with diabetes are the presence of poor glycemic control, obesity, dyslipidemia, and hypertension. In recent decades, several clinical trials have investigated the effect of intensive treatment of hyperglycemia on cardiovascular risk reduction, in both T1D and T2D, like the DCCT and UKPDS, and the main lesson learned from these trials is that intensive treatment of hyperglycemia initiated early in patients with short duration of diabetes and low cardiovascular risk, result in cardiovascular benefits. The same is not true for older patients exposed to hyperglycemia for a long time and with a high cardiovascular risk profile. This protection might result from a mechanism known as “metabolic memory,” which means that the effect of the early glycemic exposure environment is imprinted in target organs resulting in long-term deleterious or protective effects. Obesity, especially with visceral fat deposition, is associated with low-grade inflammation, which plays a role in the pathogenesis of diabetes, and both diseases are associated with significant increase in morbidity and mortality due to CVD. Dyslipidemia mainly that represented by high levels of LDL-cholesterol is also a risk factor for CVD because small increases in LDL-cholesterol levels increase the risk for CVD. The coexistence of hypertension and diabetes increase the risk of developing macrovascular complications (myocardial infarction, stroke) and also microvascular complications (nephropathy and retinopathy).

 These clinical conditions might be associated with intracellular and mitochondrial metabolic changes that can result in oxidative stress, a state of low-grade inflammation characterized by overexpression of cytokines produced by adipose tissue, activated macrophages and other cells, and the presence of many inflammatory mediators that will finally cause a generalized endothelial dysfunction or even a cardiovascular autonomic neuropathy, an important cause of sudden death among subjects with diabetes. 

 The proposed mechanisms that can link accelerated atherosclerosis and increased cardiovascular risk in subjects with diabetes are still poorly understood. It has been suggested that an association between hyperglycemia and epigenetic factors by different types of reactions could be responsible for the interaction between genes and environment and for this reason account for the association between diabetes and cardiovascular disease. Many trials have shown that an early intervention in patients with short duration of diabetes could result in cardiovascular benefits, but there is no robust evidence that justify screening for subclinical atherosclerosis in asymptomatic patients with diabetes.

 The purpose of this paper was to describe the association between poor glycemic control, oxidative stress, markers of insulin resistance and of low-grade inflammation that have been suggested as putative factors linking diabetes, and cardiovascular disease and to elucidate the mechanisms involved in the pathogenesis of CVD in this population. 

## Figures and Tables

**Table 1:** Differences of the effects of glycemic control in cardiovascular risk reduction in type 2 diabetes.

	UKPDS-10 years	VADT	ADVANCE	ACCORD
Sample size	5,102*	1,791	11,140	10,251
Followup (years)	10	5.6	5	3.4
Baseline characteristics				
Age (years)	54	60.4	58	62.2
Duration of diabetes (years)	Recently diagnosed	11.5	8	10
Presence of cardiovascular disease	9%	40%	32%	35%
Presence of microvascular complications	18%	62%	10%	Albuminuria 14.0 (6.9–45.8)^#^
A1c levels	6.2%	8.3%	7.5%	8.3%
Effects of intensive treatment				
Difference in A1c levels (intensive/conventional)	7.0/7.9%	6.9/8.4%	6.5/7.3%	6.4/7.5%
Reduction in macrovascular events	Sulfa/insulin group ↓15% MI, ↓13% death Metformin group ↓33% MI, ↓27% death	NS	NS	↓Nonfatal MI ↑Death
Reduction in microvascular events (diabetic retinopathy, nephropathy, or neuropathy)	↓24% (combined)	NS	↓incident nephropathy	—

NS: nonstatistically significant.

A1c: glycated hemoglobin; MI: myocardial infarction.

*3,277 posttrial monitoring.

^#^The percentage of subjects with microvascular complications is not available. Ratio of urinary albumin (mg) to creatinine (g); median (Interquartile range).

## Abbreviations

ABPI: Ankle brachial pressure index
ACCORD: Action to Control Cardiovascular Risk in Diabetes
ACCF: American College of Cardiology Foundation
ADA: American Diabetes Association
ADVANCE: Action in Diabetes and Vascular Disease: Preterax and Diamicron Modified Release Controlled Evaluation
AGE: Advanced glycation end products
AHA: American Heart Association
ANS: Autonomic Nervous System
BARI 2D: Bypass Angioplasty Revascularization Investigation 2 Diabetes Trial
BMI: Body mass index
BP: Blood pressure
CAC: Coronary artery calcium
CAD: Coronary artery disease
CAN: Cardiac Autonomic Neuropathy
CARDS: Collaborative Atorvastatin Diabetes Study
CHF: Congestive heart failure
CIMT: Carotid intima-media thickness
CRP: C-reactive protein
CVD: Cardiovascular disease
DCCT: Diabetes Control and Complications Trial
DIAD: Detection of Silent Myocardial Ischemia in Asymptomatic Diabetic Subjects
DPP: The Diabetes Prevention Program
EDIC: Epidemiology of Diabetes Interventions and Complications Trial
ERK: Extracellular signal regulated kinase
ET-1: Endotelin-1
FFA: Free fatty acids
FIELD: Fenofibrate Intervention and Event Lowering in Diabetes
FMD: flow-mediated dilation
GSK-3: Glycogen synthases kinase-3
HATS: HDL Atherosclerosis Treatment Study
HbA1c;: Glycated hemoglobin
HDL: High-density lipoprotein
ICAM-1: Intercellular adhesion molecule 1
IDF: International Diabetes Federation;
IL-1: Interleukin 1
IL-6: Interleukin 6
IKK*β*: Inhibitor of nuclear factor kappa-B kinase subunit beta
IRS-1: Insulin receptor substrate-1
LDL: Low-density lipoprotein
MCP-1: Monocyte chemoattractant molecule 1
MMP: Matrix metalloproteinase
MPI: Myocardial perfusion imaging
mTOR: Mammalian target of rapamycin
NADPH: Nicotinamide adenine dinucleotide phosphate
NCEP ATP III: National Cholesterol Education Programme Adult Treatment Panel III
NFAT-3: Nuclear factor in activated lymphocytes
NF-*κ*B: Nuclear factor kappa-*β*
NCEP ATP III: National Cholesterol Education Program Adult Treatment Panel III Guidelines
NHANES: National Health and Nutrition Examination Survey
NO: Nitric oxide
NOO^−^: Peroxynitrite
NOS: Nitric oxide synthase
PAI-1: Plasminogen activator inhibitor-1
PDGF: Platelet derived growth factor
PKC: Protein kinase C
PPAR-*α*: Peroxisome proliferator-activated receptor *α*
PREDICT: Patients with Renal Impairment and Diabetes undergoing Computed Tomography
RNS: Reactive nitrogen species
ROS: Reactive oxygen species
sBP: Systolic blood pressure
SMI: Silent myocardial ischemia
T1D: Type 1 diabetes
T2D: Type 2 diabetes
TGF-*β*: Transforming growth factor-*β*
TNT: Treatment to new targets
tPA: Tissue plasminogen activator
UKPDS: United Kingdom Prospective Diabetes Study
WHO: World Health Organization
VADT: Veterans Affairs Diabetes Trial
VCAM-1;: Vascular cell adhesion molecule-1
VEFG: Vascular endothelial growth factor
VLDL: Very low-density lipoprotein
VSMC: Vascular smooth muscle cells.
